# An Advertisement and Article Analysis of Skin Products and Topics in Popular Women’s Magazines: Implications for Skin Cancer Prevention

**DOI:** 10.15171/hpp.2015.031

**Published:** 2016-01-30

**Authors:** Corey H. Basch, Jennifer Mongiovi, Grace Clarke Hillyer, MD Fullwood, Danna Ethan, Rodney Hammond

**Affiliations:** ^1^Department of Public Health, William Paterson University, Wayne, NJ 07470, USA; ^2^Department of Epidemiology, Mailman School of Public Health, Columbia University NY, NY 10032, USA; ^3^Health and Behavior Studies, Teachers College, Columbia University, NY, NY 10027, USA; ^4^Department of Health Sciences, Lehman College, The City University of New York, USA; ^5^Department of Health and Nutrition Sciences, Montclair State University, USA

**Keywords:** Advertisement, Skin, Female, USA

## Abstract

**Background:** In the United States, skin cancer is the most commonly diagnosed cancer, with an estimated 5 million people treated per year and annual medical treatment expenditures that exceed 8 billion dollars. The purpose of this study was two-fold: 1) to enumerate the number of advertisements for skin products with and without Sun Protection Factor (SPF) and to further analyze the specific advertisements for sunblock to determine if models, when present, depict sun safe behaviors and 2) to enumerate the number of articles related to the skin for content. Both aims include an assessment for differences in age and in magazines targeting a Black or Latina population.

**
Methods:** The sample for this cross sectional study was comprised of 99 issues of 14 popular United States magazines marketed to women, four of which market to a Black or Latina audience.

**
Results:** There were 6,142 advertisements, of which 1,215 (19.8%, 95% CI: 18.8-20.8%) were related to skin products. Among the skin product advertisements, 1,145 (93.8%, 95% CI: 93.9-96.3%) depicted skin products without SPF. The majority of skin articles (91.2%, 95% CI: 91.7-100.0%), skin product advertisements (89.9%, 95% CI: 88.2-91.6%), and sunblock advertisements featuring models (were found in magazines aimed at the older (>24 yr) audience.

**
Conclusion:** Future research on this topic could focus on the extent to which images in these magazines translate into risky health behaviors, such as sun seeking, or excessive other harmful effects of UV radiation.

## Introduction

In the United States, skin cancer is the most commonly diagnosed cancer, with an estimated 5 million people treated per year and annual medical treatment expenditures that exceed 8 billion dollars.^1^ Of the three types of skin cancer including basal cell, squamous cell, and melanoma; basal and squamous cell carcinomas are less deadly forms of skin cancer with 3.5 million cases diagnosed per year and an estimated 2,000 resultant deaths.^2^ Melanoma, considered to be more aggressive form of skin cancer, is more likely to be fatal.^3^ There are over 60,000 cases of melanoma diagnosed each year, resulting in 9,000 deaths from this form of skin cancer annually.^1^

Over the past 30 years, incidence rates for melanoma have increased rapidly, particularly among adolescents and young adults.^4,5^ Individuals of all races are susceptible to skin cancer but Caucasians are at higher risk^6^ and have the highest incidence (25/100,000) followed by Hispanics (4/100,000), and Blacks (1/100,000).^7^Unfortunately, diagnoses occur at later stages for people of color when the disease is more advanced and less amenable to treatment.^8^

Of the numerous risk factors associated with skin cancer (family history, being pale skinned, having blonde or red hair and blue or green eyes^9^), the factor most easily controlled is exposure to ultraviolet radiation (UVR)^10^ from the sun or from artificial tanning that causes cellular damage and even genetic mutations.^11^ Hence, it is recommended that sunscreen be used and artificial tanning be avoided.^12^ Yet, despite this urgent public health concern, there has been a decline in the percentage of youth who wear sunscreen, and artificial tanning behaviors remain a problem in some subsets of the population.^13^ Sunscreen use also remains low in adults^14^, and artificial tanning rates are highest (32%) in non-Hispanic white women aged 18-21 yr.^15^

The influence of content contained in magazines and leverage of mass media have potential to sway how the public perceives information about skin cancer and tanning information.^16^ A study aimed at determining the frequency of risk factors and ultraviolet (UV) messages displayed in 20 popular US men and women magazines found both articles and magazines encouraged sunscreen behavior but helpful prevention and risk factor information was infrequent in the images.^16^ Another study focusing on products devoted to skin health in popular women’s health and fitness found only 2.9% of articles related to skin health and only 20% of skin products had sun protection factor.^17^

In concert, two popular parenting magazines were analyzed during months of peak ultraviolet radiation exposure to capture sun protection content and to examine skin products advertised with or without sun protective factor (SPF).^18^ Again, the focus on SPF was low despite the pervasive amount of skin product advertisements in these magazines.^18^ These findings are in agreement with other studies that highlight how skin cancer and tanning content may diminish the risks and prevention associated with skin cancer for public understanding.^16^ Despite the existing research on this topic, there is a gap in the literature regarding what is depicted in sunblock ads in women’s magazines of varied readership characteristics.

The purpose of this study was two-fold: 1) to enumerate the number of advertisements for skin products with and without SPF and to further analyze the specific advertisements for sunblock to determine if models, when present, depict sun safe behaviors and 2) to enumerate the number of articles related to the skin for content. Both aims include an assessment for differences in age and in magazines targeting a Black or Latina population.

## Materials and Methods

The sample for this cross sectional study was comprised of a convenience sample of 99 issues of fourteen popular U.S. magazines marketed to women, four of which market to a Black or Latina audience. The following magazines were included in the sample Allure, Cosmopolitan, Elle, Essence, Girl’s Life, Glamour, Marie Claire, Seventeen, Teen Vogue, and Vogue and Cosmo Latina, Ebony, Jet, Latina which are specifically marketed to a Black or Latina audience. The selected issues were published between the months of January to August 2014, the months when skin and skin protection would most likely be discussed. It is important to note that 12 issues were seasonal and spanned across more than one month. The selection of these magazines was based on their high collective readership of nearly 80 million women and their focus on beauty-related issues.^19-31^ The mean age for readership of these magazines was 34.8 years and is highly relevant as rates of skin cancer are rapidly increasing in those under 40.^32^

From prior studies of skin editorials and products, a coding sheet was adapted.^17,33^ All articles were enumerated and categorized for content from the table of contents of each magazine. Letters to the editor were not included in the editorial count. If an article related to skin was identified, it was read in-depth and a content analysis was performed on the information covered in the article. Specifically, it was determined if the article content covered any of the following areas: avoidance of sun in peak hours, cancer prevention, use of sunscreen, use of protective clothing, use of protective eyewear, use of sunless tanner, dangers of tanning, benefits of tanning, concealing wrinkles, or other positive sun-avoidant behaviors.

All paid advertisements and those that were permanent parts of the magazine, including the back cover, were evaluated. Excluded from our analysis were materials that could be torn out of the magazine, “staff picks” or item highlights, as well as front covers. The total number and number of advertisements related to skin products was determined. Skin product advertisements were grouped as either having SPF or without SPF. Within each group products were coded as: anti-aging, anti-wrinkle, cleanser, makeup/foundation, moisturizer, sunless tanner, or other. Sunblock was recorded separately. When advertisements related to sunblock were identified, it was determined if models were present and if they exhibited the following: 1) being covered in protective clothing, 2) wearing hats, 3) wearing sunglasses 4) are in the shade, and 5) other. For each magazine, the median age of the readership was gathered from online press kits.

This descriptive analysis included determination of frequencies and range of frequency. Median magazine readership was dichotomized as less than or equal to 24 and greater than 24 years of age. The relationship between the skin articles and skin product advertisements and age of the readership and whether the magazine was marketed to a Black or Latina population (yes/no) was ascertained using Chi square analysis. *P-*values <0.05 were considered statistically significant. Analyses were conducted using IBM SPSS version 22 (Chicago, IL, USA). To determine inter-rater reliability, skin items in 10% of the magazines were re-coded and measured using Cohen’s Kappa. Intra-rater reliability was found to be excellent at 0.85.

### Ethical Issues

In terms of ethical aspects, studies that do not include human subjects are not reviewed by The Institutional Review Boards at William Paterson University and Lehman College.

## Results

A total of 2,528 articles were identified with 154 (6.1%, 95% CI: 5.2-7.0%) related to health (Table 1). Of these health-related articles, 34 were about skin (22.1%, 95% CI: 26.5-41.5%). Topics in these skin articles included: avoidance of sun in peak hours (n=8; 23.5%, 95% CI:9.3-37.8%), cancer prevention (n=6; 17.6%, 95% CI: 4.8-30.4%), use of sunscreen (n=11; 32.4%, 95% CI:16.7-48.1%), use of protective clothing (n=3; 8.8, 95% CI: 0.0-18.3%), use of protective eyewear (n=5; 14.7%, 95% CI: 2.8-26.6%), dangers of tanning (n=4; 11.8%, 95% CI: 1.0-22.6%), concealing wrinkles (n=3; 8.8, 95% CI:0.0-18.3%), and other topics such as skin care routines, makeup application, and acne (n=20; 58.8%, 95% CI: 42.3-75.3%). No articles mentioned using sunless tanner or the benefits of tanning.

There were 6,142 advertisements, of which 1,215 (19.8%, 95% CI: 18.8-20.8%) were related to skin products (Table 1).Among the skin product advertisements, 1,145 (93.8%, 95% CI: 93.9-96.3) depicted skin products without SPF. They were dispersed among the following products: anti-aging cream (n=148; 12.9%, 95% CI: 11.0-14.8%), anti-wrinkle (n=32; 2.8%, 95% CI: 1.9-3.8%), cleanser (n=213; 18.6%, 95% CI: 16.4-20.9%), makeup/foundation (n=492; 43.0%, 95% CI: 40.1-45.9%), moisturizer (n=195; 17.0, 95% CI: 14.8-19.2%), and sunless tanner (n=5; 0.4%, 95% CI: 0.0-0.8%). Conversely, there were only 32 ads for skin products with SPF, which included the following products: anti-aging cream (n=1; 3.1%, 95% CI: 0.0-9.1%), anti-wrinkle (n=1; 3.1%, 95% CI: 0.0-9.1%), cleanser (n=1; 3.1%, 95% CI:0.0-9.1%), foundation (n=8; 25.0%, 95% CI: 10.0-40.0%), and moisturizer (n=21; 65.6%, 95% CI:49.1-82.1%).There were a total of 38 advertisements for sunblock. In the 15 ads that included models, only 4 were covered in protective clothes (26.7%, 95% CI: 4.3-49.1%), one had a hat (6.7%, 95% CI: 0.0-19.4%), 5 were wearing sunglasses (33.3%, 95% CI: 9.5-57.2%), and none were in the shade.

**Table 1:** Total skin articles in fourteen popular U.S. magazines marketed to women, January through August, 2014 (n = 99)

	**n (%)**	**Range per** **magazine issue**
**Total articles**	2,528	11-50
Health related topics^a^	154 (6.1)	0-5
Skin-related	34 (22.1)	0-2
**Skin-related article topics**		
Avoidance of sun in peak hours^b^	8 (23.5)	0-1
Cancer prevention^b^	6 (17.6)	0-1
Use of sunscreen^b^	11 (32.4)	0-1
Use of protective clothing^b^	3 (8.8)	0-1
Use of protective eyewear^b^	5 (14.7)	0-1
Use of sunless tanner^b^	0 (0.0)	-
Dangers of tanning^b^	4 (11.8)	0-1
Benefits of tanning^b^	0 (0.0)	-
Concealing wrinkles^b^	3 (8.8)	0-1
Other	20 (58.8)	0-3
**Total Advertisements**	6, 142	2-142
Skin product advertisements^a^	1,215 (19.8)	0-34
Skin advertisements with SPF^c^	32 (2.7)	0-3
Anti-aging	1 (3.1)	0-1
Anti-wrinkle	1 (3.1)	0-1
Cleanser	1 (3.1)	0-1
Makeup/Foundation	8 (25.0)	0-1
Moisturizer	21 (65.6)	0-1
Sunless tanner	0 (0.0)	--
Other	2 (6.3)	0-2
Skin advertisements without SPF^c^	1,145 (95.1)	0-34
Anti-aging	148 (12.9)	0-8
Anti-wrinkle	32 (2.8)	0-3
Cleanser	213 (18.6)	0-8
Makeup/Foundation	492 (43.0)	0-10
Moisturizer	195 (17.0)	0-7
Sunless tanner	5 (0.4)	0-1
Other	60 (5.2)	0-3
Sunblock^c^	38 (3.1)	0-6
**Sunblock advertisements featuring models** ^d^	15 (39.5)	0-3
Being covered in protective clothing	4 (26.7)	0-2
Wearing hats	1 (6.7)	0-1
Wearing sunglasses	5 (33.3)	0-1
Are in the shade	0 (0.0)	-

^a^Percentage based on all articles or advertisements ^b^Groups not mutually exclusive ^c^ Percentage based on all skin product advertisements ^d^Percentage of sunblock advertisements

Table 2 displays an analysis of skin article content and skin product advertisement stratified by median readership age (≤24 vs. >24 yrs) and by whether or not the magazine was targeted toward a Black and Latino market. The majority of skin articles (91.2%, 95% CI: 91.7-100.0%), skin product advertisements (89.9%, 95% CI: 88.2-91.6%), and sunblock advertisements featuring models (were found in magazines aimed at the older (>24 yrs) audience. By age of the readership, the only difference found was in the number of sun products with SPF representing 2.8% (95% CI: 0.0-8.5%) of all skin product advertisements in magazines geared toward women >24 years opposed to 0.8% (95% CI: 0.0-4.5%) in magazines for younger women (*P*= 0.185*).*Sunblock advertisements were found most often in magazines for the older readership and none of the advertisements with models in the magazines for younger women displayed any sun protective behaviors. With regard to differences in skin articles and skin product advertising in magazines directed at Black and Latina audiences versus the general population of women, more skin articles (82.4%, 95% CI: 69.6-95.2% vs. 17.6%, 95% CI: 4.8-30.4%) and skin product (85.2%, 95% CI: 83.2-87.2% vs. 14.8%, 95% CI: 12.8-16.8%) were found. Sunblock advertisements with models were found exclusively in magazines for the general population of women with no such advertisements in magazines targeting women of color. Among skin articles, avoidance of the sun during peak hours and use of both protective clothing and eyewear were discussed only in articles in magazines not marketed to the Black and Latina populations.

**Table 2:** Audience of skin articles and product advertisements in fourteen popular U.S. magazines marketed to women, January through August, 2014 (n = 99)

	**Median readership age ≤ 24**	**Median readership age > 24**	***P*** **-value**	**Not marketed to Black or Latina** **audience**	**Marketed to Black or** **Latina** ** audience**	***P*** **-value**
	**n (%)**	**n (%)**		**n (%)**	**n (%)**	
**Skin articles**	3 (8.8)	31 (91.2)	--	28 (82.4)	6 (17.6)	--
Avoidance of sun in peak hours	1 (33.3)	7 (22.6)	0.675	8 (28.6)	0 (0.0)	0.134
Cancer prevention	0 (0.0)	6 (19.4)	0.382	5 (17.9)	1 (16.7)	0.885
Use of sunscreen	1 (33.3)	10 (32.3)	0.876	8 (28.6)	3 (50.0)	0.816
Use of protective clothing	0 (0.0)	3 (9.7)	0.573	3 (10.7)	0 (0.0)	0.401
Use of protective eyewear	0 (0.0)	5 (16.1)	0.451	5 (17.9)	0 (0.0)	0.262
Use of sunless tanner	0 (0.0)	0 (0.0)	--	0 (0.0)	0 (0.0)	--
Dangers of tanning	0 (0.0)	4 (12.9)	0.508	4 (14.3)	0 (0.0)	0.324
Benefits of tanning	0 (0.0)	0 (0.0)	--	0 (0.0)	0 (0.0)	--
Concealing wrinkles	1 (33.3)	2 (6.5)	0117	2 (7.1)	1 (16.7)	0.455
Other sun avoidant behaviors	1 (33.3)	18 (58.1)	0.410	15 (53.6)	4 (66.7)	0.558
**Skin Product Advertisements**	123 (10.1)	1,092 (89.9)	--	1,035 (85.2)	180 (14.8)	--
Skin advertisements with SPF^b^	1 (0.8)	31 (2.8)	0.185	27 (2.6)	5 (2.7)	0.923
Sunblock	3 (2.4)	35 (3.2)	0.644	38 (3.7)	0 (0.0)	0.009
**Sunblock advertisements featuring models** ^c^	3 (100.0)	12 (34.3)	0.025	15 (39.5)	0 (0.0)	--
Being covered in protective clothing	0 (0.0)	4 (33.3)	0.243	4 (26.7)	0 (0.0)	--
Wearing hats	0 (0.0)	1 (8.3)	0.605	1 (6.7)	0 (0.0)	--
Wearing sunglasses	0 (0.0)	5 (41.7)	0.171	5 (33.3)	0 (0.0)	--
In the shade	0 (0.0)	0 (0.0)	--	0 (0.0)	0 (0.0)	**--**

## Discussion

Given the high incidence and prevalence of skin cancer, it is important that sun protective behaviors be incorporated into mainstream print materials regardless of the target audience. More articles focus on covering up damaged skin than preserving healthy skin. The findings of this study are interesting for several reasons. First, nearly all of the products advertised for skin did not include SPF, which is recommended to decrease harmful UVB rays. The aforementioned study of advertisements in parenting magazines drew similar conclusions.^18^

Secondly, we found that when models were present in sunblock advertisements, they rarely exhibited use of sun protective clothing. In fact, none of the advertisements with models in the magazines for younger women displayed any sun protective behaviors. This finding is concerning, as initiating sun protective behaviors at a young age can be very beneficial in skin cancer prevention efforts^34^ as melanoma known to be the most common cancer in women aged 15-29 years.^35^ Researchers have noted a similar finding regarding protective clothing. For example, a study involving a longitudinal content analysis of women’s magazines found preventive methods of coverage focused more on sunscreen, but rarely promoted more important methods such as wearing clothing that could protect one from the harmful effects of the sun.^36^

Third, results demonstrated that sunblock advertisements were found most often in magazines for the older readership. Again, with advocates stating that sun protective behaviors begin at a young age, the images displayed in these magazines can result in conflicting messages to youth. A similar conflicting message was found in an Australian study, whereby younger Caucasian females in popular magazines displayed darker tan and more exposed body parts in beach and pool advertisements. Moreover, 89% of 4,949 images were of un-shaded female models minus sun protective behaviors exhibited such as hats.^37^

Future research on this topic could focus on the extent to which images in these magazines translate into risky health behaviors, such as sun seeking, or excessive tanning. Around the world, efforts are being made to decrease rates of melanoma and other harmful effects of UV radiation. An example is enacting legislation to restrict minors from indoor tanning.^38^ Australia’s Slip Slop Slap! Campaign has heightened awareness about the use of sunblock during times of UV exposure.^39^ While the responsibility of editors of magazines is to ensure the accuracy and suitability of the content in their publications, heightening their awareness of the influence of negative health messages promoted in advertising may lead to greater sensitivity to the impact of these messages on readership attitudes and behaviors.

## Conclusion

Media channels such as magazines could play a great role in helping to reduce rising rates of skin cancer by portraying images consistent with recommendations for risk reduction from UV radiation, namely by way of sun protection.

## Acknowledgements

There was no funding for this study. The authors declare that there is no conflict of interests.

## Conflict of interest

None of the authors report a conflict of interest.
